# Biomarker Development for Metastatic Renal Cell Carcinoma: Omics, Antigens, T-cells, and Beyond

**DOI:** 10.3390/jpm10040225

**Published:** 2020-11-13

**Authors:** Benjamin Miron, David Xu, Matthew Zibelman

**Affiliations:** Department of Hematology/Oncology, Fox Chase Cancer Center, Philadelphia, PA 19111, USA; benjamin.miron@tuhs.temple.edu (B.M.); david.xu@tuhs.temple.edu (D.X.)

**Keywords:** biomarkers, renal cell carcinoma, clear cell, VEGF, immunotherapy, PD-L1, immune checkpoint inhibitors, immune checkpoint blockade, tyrosine-kinase inhibitors

## Abstract

The treatment of metastatic renal cell carcinoma has evolved quickly over the last few years from a disease managed primarily with sequential oral tyrosine kinase inhibitors (TKIs) targeting the vascular endothelial growth factor (VEGF) pathway, to now with a combination of therapies incorporating immune checkpoint blockade (ICB). Patient outcomes have improved with these innovations, however, controversy persists regarding optimal sequence and patient selection amongst the available combinations. Ideally, predictive biomarkers would aid in guiding treatment decisions and personalizing care. However, clinically-actionable biomarkers have remained elusive. We aim to review the available evidence regarding biomarkers for both TKIs and ICB and will present where the field may be headed in the years to come.

## 1. Introduction

**Figure 1:**
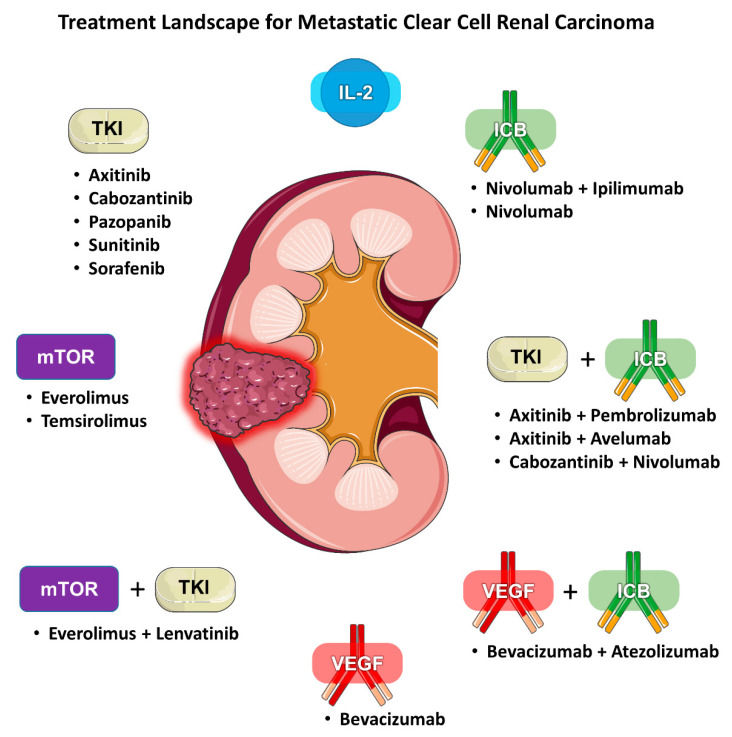
Treatment landscape for metastatic clear cell renal carcinoma.

Renal cell carcinoma (RCC) is traditionally classified according to its histology. Clear cell (ccRCC) is the most common subtype, accounting for 75–85% of all RCCs. Current first-line standard of care therapies for metastatic ccRCC involve the use of vascular endothelial growth factor (VEGF) inhibitors, checkpoint inhibitors, anti-CTLA4 agents, or a combination of these drugs. Choice of therapy is guided by whether the patient’s disease falls under favorite or intermediate/poor risk based on validated prognostic models. Within each risk category, there are several acceptable alternatives, including VEGF inhibitor monotherapy, combination immunotherapy (e.g., ipilimumab/nivolumab), or a combination of a VEGF inhibitor and a checkpoint inhibitor (e.g., axitinib/pembrolizumab). Given the increasing number of available treatment options for mRCC there is also a growing need for predictive biomarkers to help guide clinicians (Figure 1). We aim to review the literature regarding the evidence for selecting one type of regimen over another and determining who would benefit more from either angiogenesis antagonism or immune checkpoint blockade (ICB).

## 2. Biomarkers for Angiogenesis Inhibitors

### 2.1. International Metastatic Renal Cell Carcinoma Database Consortium Score

Past attempts to create clinical prognostication tools to risk stratify patients treated with molecularly-targeted agents include the International Metastatic Renal Cell Carcinoma Database Consortium (IMDC) score. This was a model based on a multicenter study of 645 patients with metastatic RCC (mRCC) who were treated with VEGF pathway-targeted therapies, such as sunitinib, sorafenib, or bevacizumab (plus interferon-alfa) for mRCC [1]. Six factors were noted to be associated with worse survival: Karnofsky Performance Status (KPS) score < 80, time from diagnosis to initiation of targeted therapy < 1 year, hemoglobin less than lower limit of normal, corrected calcium greater than the upper limit of normal, absolute neutrophil count greater than upper limit of normal, and platelet count greater than the upper limit of normal. Scoring was binary with each factor assigned a score of 0 or 1, and a total sum was taken. A total score of 0 corresponded to a favorable risk group with a median overall survival (mOS) of 43.2 months; a score of 1–2 indicated intermediate risk with a mOS of 22.5 months; and a score ≥ 3 represented a poor risk group with a mOS of 7.8 months. This clinical prediction tool was subsequently externally validated in a study of 849 patients with mRCC who were treated with first-line anti-VEGF therapies [2]. Another study found that the IMDC score can be applied to patients who progressed after first-line anti-VEGF therapy [3]. Although there have been other risk models that have been developed, including the MSKCC model, Cleveland Clinic Foundation model, French model, and International Kidney Cancer Working Group, the IMDC model has become the one most widely utilized in contemporary clinical trials. 

While clinical tools such as the IMDC score were originally developed to estimate patients’ prognoses regardless of the treatment early in the era of VEGF-targeted therapy, there has been a gradual evolution toward assuming a role in predicting response to therapy. The IMDC criteria has been used to retrospectively risk stratify patients who underwent ICB combination therapies (e.g., ipilimumab/nivolumab vs. axitinib + pembrolizumab/avelumab) or anti-VEGF agents in first-line or second-line settings [4]. There is growing evidence that using the risk score may be useful in guiding the selection of a particular therapy. For example, in KEYNOTE-426, there was a benefit for pembrolizumab/axitinib over sunitinib in the first-line treatment of advanced ccRCC in an updated analysis [5]. However, this effect was less pronounced for patients who fell into the IMDC favorable risk category. Similarly, a prospective trial comparing ipilimumab/nivolumab (ipi/nivo) combination vs. sunitinib in untreated advanced ccRCC showed that patients who were at intermediate or poor risk and treated with ipi/nivo had superior 18-month OS, progression free survival (PFS), overall response rate (ORR), and complete response (CR) rates as compared to patients treated with sunitinib alone [6]. However, an exploratory analysis of favorable risk patients, who were found to have a lower baseline PD-L1 expression level when compared to higher risk groups, failed to demonstrate the same benefits of ipi/nivo over sunitinib. In fact, the 18-month OS trended higher for sunitinib, and the ORR was lower and mPFS shorter for the ipi/nivo group in a statistically significant manner, although there was no OS advantage for sunitinib. Given such a disparate therapeutic response to ICB vs. sunitinib based on the risk profile, it is conceivable that IMDC scoring can be used to predict treatment response in addition to estimating the prognosis. That higher risk group patients with advanced ccRCC responded better to ICB-based therapies suggests that there may be differences in the underlying tumor and/or microenvironment biology that influence response to the current available treatments. 

### 2.2. Genomic Alterations

Earlier studies on biomarkers focused on driver mutations, epigenetic modifications, or chromosomal aberrations associated with ccRCC to evaluate their potential in clinical prognostication. An obvious candidate was the *von Hippel-Lindau* (*VHL*) gene, which is inactivated in RCC via a point mutation or through epigenetic silencing. This mutation is present in about 60–90% of cases of ccRCC. Inactivation of the protein product of *VHL* leads to abnormal stabilization of hypoxia-inducible factor (HIF), which drives oncogene transcription. However, no clear relationship between *VHL* abnormalities and patient outcome exists [7]. A meta-analysis from 2017 revealed that *VHL* was not a predictive marker in patients treated with anti-VEGF-targeted agents, as abnormal *VHL* failed to show a relationship with ORR, PFS, or OS [8]. *Polybromo 1*, (*PBRM1*), also known as BAF180, is encoded in a gene locus near *VHL* and is a component of the PBAF complex, a mammalian SWItch/Sucrose Non-Fermentable (SWI/SNF) complex, which is a tumor suppressor protein that is thought to become mutated early in RCC pathogenesis [9]. Older studies evaluating its role as a prognostic marker did not include targeted agents and failed to predict cancer-specific survival [9,10]. However, a study published in 2016 showed that among 31 metastatic ccRCC patients, the vast majority of whom received anti-VEGF therapy, those who were maintained for longer durations of therapy were more likely to harbor a *PBRM1* mutation [11]. A separate study found that a group of so-called “extreme responders,” defined as partial response (PR) or complete response (CR) for ≥3 years in mRCC on either first-line sunitinib or pazopanib, was enriched for *PBRM1* mutations [12]. A third biomarker that has been studied is *SETD2*, which is sometimes co-mutated with *PBRM1*. It encodes for a histone-lysine N-methyltransferase and acts as a chromatin regulator. It does not appear to have a definite correlation with survival but is associated with higher risk of disease recurrence after surgery for localized disease. Its mutation status did not seem to correlate with PFS in mRCC patients treated with sunitinib [13].

### 2.3. Targets of Tumor-Driven Angiogenesis

With growing evidence that tumor and microenvironmental biology could impact clinical factors and lead to differential outcomes depending on the type of systemic therapy, identifying predictive biomarkers became an increasing focus. Earlier studies acknowledged the hyperangiogenic state of ccRCC and the mechanism of action of VEGF-targeted tyrosine kinase inhibitors (TKIs) as a rationale for studying the components of the VEGF signaling cascade. One study looked at whether VEGF expression in tumor and endothelial cells was predictive and/or prognostic in 41 patients with metastatic RCC (mRCC) treated with radical nephrectomy and sunitinib [14]. In this study, higher VEGF expression within the tumor cells correlated with the MSKCC group and was associated with higher tumor stage and inferior OS. There was no correlation between intratumoral VEGF expression and PFS or OS on first-line sunitinib, suggesting that the VEGF level may be prognostic but not predictive. A different study evaluated the role of serum VEGF levels in predicting treatment response to sunitinib in 85 patients with advanced RCC (mostly clear cell) who overwhelmingly fell into the favorable or intermediate categories based on the MSKCC model; these patients were undergoing systemic treatment in the second line and beyond [15]. The patients who had serum VEGF levels higher than reference value of 707 pg/mL had a longer PFS by about six months. A third small, single-institution study of 23 mRCC patients attempted to associate tumor expression of 16 selected biomarkers with treatment response to second-line sunitinib after the failure of first-line interferon-α. They quantified biomarker expression using qRT-PCR and categorized tumor response using the RECIST criteria. The authors noted that specific soluble VEGF isoforms, VEGF_121_ and VEGF_165_ in particular, were associated with partial response, and they proposed that a ratio of VEGF_121_/VEGF_165_ of <1.25 predicted superior OS [16]. Despite these studies, neither peritumoral nor serum VEGF is routinely measured in clinical practice.

### 2.4. Gene Expression Signatures

More recent studies have investigated gene expression signatures as potential guides for the tailoring of therapy. A study published in 2013 found that tumor upregulation of the so-called VEGF-dependent vascular gene profile appeared to predict improved PFS when bevacizumab was added to standard oxaliplatin-based systemic therapy for treatment-naïve metastatic colorectal cancer [17]. To see if such a phenomenon was also applicable to mRCC, an exploratory analysis of the IMmotion150 study looked at tumor mutation burden as well as angiogenesis and immune gene expression signatures in previously untreated mRCC patients who were treated with sunitinib vs. atezolizumab with or without bevacizumab [18]. The Angio gene signature consisted of the following genes: *VEGFA*, *KDR*, *ESM1*, *PECAM1*, *ANGPTL4*, and *CD34*. In the IMmotion150 study, increased the expression of the Angio signature correlated with a higher ORR and longer PFS among the group of patients treated with sunitinib, including a 7% CR rate as compared to 0% in the low Angio expression group. If the Angio expression level was low, the combination arm had better PFS as compared to that of sunitinib monotherapy. Although the angiogenesis gene signature tended to have upregulation of *VHL* and *PBRM1* mutants, *VHL* status itself was not associated with differences in PFS [18]. Although these results were intriguing, they remain hypothesis-generating. 

### 2.5. Association of Angiogenesis Signatures with Traditional Biomarkers

One group analyzed data from the phase III COMPARZ trial to associate tumor gene expression profiling with clinical endpoints in untreated metastatic ccRCC [19]. This trial randomized untreated mRCC patients to receive either pazopanib or sunitinib to assess for differences in efficacy, toxicity, and quality of life. In patients treated with an anti-VEGF TKI, such as sunitinib or pazopanib, increased expression of angiogenesis genes significantly correlated with better ORR, PFS, and OS as compared to those with lower expression. However, this benefit appeared to be abrogated in the group of patients that was enriched for *TP53* and *BAP1* mutations. The group of patients with higher frequency of *TP53* and *BAP1* mutations tended to have high immune infiltration and higher PD-L1 expression. Ultimately, there was no significant difference in the angiogenesis gene profile among the three different IMDC risk groups, suggesting that the prediction of enhanced response to TKIs was independent of previously established clinical prognostic markers. As was the case in IMmotion150 study, in the COMPARZ trial, mRCC tumors with *PBRM1* mutations were noted to have upregulated angiogenesis gene expression in contrast to tumors harboring *BAP1* mutations, which were associated with decreased expression of angiogenesis-related genes. There was no association between the angiogenesis gene signature expression level and *SETD2* mutation. 

### 2.6. Pure VEGF Antagonism vs. Combination Anti-VEGF/ICB Therapy

Combination therapy with anti-angiogenic agents and ICB has become the standard of care for the majority of patients with mRCC. It would be clinically actionable to understand whether angiogenic or immune biomarkers could help predict the therapeutic response in order to better stratify patients to combination therapy versus a single agent strategy to minimize toxicity while optimizing efficacy. A study evaluated association between angiogenesis signatures and outcomes in the phase 3 JAVELIN Renal trial that enrolled patients with untreated mRCC and randomized them to avelumab/axitinib or sunitinib. High levels of expression of angiogenesis-related genes were associated with better PFS in patients treated with sunitinib. In patients whose tumors had low expression of an angiogenesis signature, there was improved PFS in patients treated with avelumab/axitinib in comparison to sunitinib [20]. All in all, several studies have shown evidence that upregulation of a set of angiogenesis-related genes seemed to help predict a better response to anti-VEGF therapy, which would be important information in guiding selection of therapy. Those who registered low in angiogenesis gene expression predictably did not benefit as much, and there is a suggestion that mRCC patients harboring low tumor angiogenesis gene expression signatures represent an immune-enriched subtype that is less likely to respond to anti-VEGF therapy alone and may benefit more from strategies involving ICB. 

### 2.7. Predictive Value of Trends in Angiogenesis-Related Biomarkers during Treatment

A retrospective analysis of tumor samples or blood samples obtained from 52 mRCC patients treated with first-line axitinib/pembrolizumab in a phase Ib trial was assessed for angiogenesis-related biomarkers [21]. Angiopoietin-1 and 2 (Ang-1; Ang-2), VEGF, VEGFR-1, VEGFR-2, and VEGFR-3 were chosen for evaluation as serum biomarkers given their known roles in angiogenesis [22]. The study authors found that serum concentrations of Ang-1, Ang-2, VEGF, VEGFR2, and VEGFR3 at baseline had no correlation with PFS as a continuous variable. However, when patients were divided into two categories of PFS (<9 months vs. >20 months), the median Ang-2 protein level on treatment was lower in the PFS > 20 month group as compared to PFS < 9 months. The Ang-1 protein level was lower in patients with PFS > 20 months at the end of the treatment. The ratio of VEGF at the end of treatment to VEGF at baseline was also lower for patients who experienced PFS > 20 months [21]. This study suggested the potential that these biomarkers may have in assessing whether a patient on treatment is a responder, enabling earlier escalation of therapy for those with a lower chance of response. These results require prospective validation, however (Table 1).

## 3. Biomarkers for Immunotherapy

Renal cell carcinoma is often considered an immunogenic tumor. This has been evidenced from pathologic examination of RCC tumor tissue showing significant infiltration by both T-cells and natural killer cells [23]. In addition, efficacy of early immunotherapy agents, like interleukin-2 (IL-2) and Interferon alpha (IFN-α), and more recently ICB in the treatment of RCC support this notion pragmatically [24,25,26]. ICB targeting the programmed death 1 (PD-1) and cytotoxic T-lymphocyte associated protein 4 (CTLA-4) pathways have demonstrated favorable outcomes, with ORRs of 25% for anti-PD-1 targeted single-agent therapy and up to 39% and 59% when combined with CTLA-4 or vascular endothelial growth factor (VEGF) inhibitors, respectively [5,6,27]. Consequently, combination strategies with ICB have become the standard of care for most eligible mRCC patients. 

However, since both combination ICB/ICB and ICB/TKI regimens are approved as first-line therapy for mRCC, it would be beneficial to have clinical biomarkers to understand which tumors are more likely to benefit from an immunotherapy based regimen versus a combination regimen with VEGF inhibition. 

### 3.1. PD-L1 Expression

The programmed death-ligand 1 (PD-L1), also known as B7 homolog 1 (B7-H1), is found on tumor and immune cells in the TME, and its receptor PD-1 on T-cells are the primary targets for this form of ICB. In the era of ICB, expression of PD-L1 by immunohistochemistry (IHC) has been a focus of much of biomarker research across tumor types but in the case of mRCC it has not borne out to be a very useful predictive biomarker. 

When focusing specifically on registration studies for ICB in mRCC, patients without any measurable PD-L1 expression have benefited from these drugs. In a meta-analysis of six randomized controlled trials of ICB in mRCC an association was observed between PD-L1 expression and PFS, but the analysis failed to show significant correlation with OS [28]. The authors concluded from this data that the role of PD-L1 expression in selecting treatment for RCC was not well established, in line with FDA drug approvals and the NCCN guidelines which do not include or require PD-L1 expression [29,30]. This difference is likely multifactorial and could be due to the unique biology of RCC, related to the non-standardized testing utilized in for PD-L1 expression as a biomarker in earlier trials, including the use of different antibodies for various IHC assays and inconsistent cutoffs for positivity, tumor heterogeneity and the dynamic nature of PD-L1 expression on tumor cells [31]. 

Furthermore, prior to the era of immunotherapy, PD-L1 expression by IHC was studied in mRCC and was shown to be associated with poor prognosis [32]. The observation that PD-L1 positivity is linked to poor prognosis was again reported more recently in a post-hoc analysis of the COMPARZ trial (pazopinib vs. sunitinib) which showed that patients who were PD-L1 positive had significantly worse OS and PFS compared to the PD-L1 negative population. This is also supported by an analysis of CHECKMATE-214 study (nivolumab+ipilumimab vs. sunitinib) which demonstrated that PD-L1 positivity was more common in patients with intermediate and poor risk disease as defined by IMDC criteria compared to those with favorable risk disease [6]. It is possible that the prognostic implications of PD-L1 positivity in mRCC also have a negative impact on its usefulness as a predictive biomarker.

### 3.2. Genomic Markers

#### 3.2.1. PBRM1 Mutations

Differences in the genomic landscape of RCC have also been the subject of much study in the search for clinical biomarkers for ICB treatment PBRM1 and PBAF complex mutations have drawn much attention in this regard and, as discussed above, have also been investigated as both a prognostic and predictive markers for VEGF TKIs. In relation to ICB, PBRM1 was first identified by Miao et al. in a set of 35 patients with mRCC who participated in a prospective clinical study of nivolumab. Whole-exome sequencing was performed on tissue samples and identified PBRM1 as being strongly enriched in the group who derived clinical benefit. This finding was then validated in a separate 63 patient cohort treated with PD-1 or PD-L1 inhibitions alone or in combination with anti-CTLA-4 therapies and replicated findings of association with clinical benefit [33]. 

However, after this initial publication, PBRM1 mutations were subsequently studied in several additional patient cohorts. An analysis by McDermott et al. of a first-line clinical trial of atezolizumab alone or in combination with bevacizumab vs. sunitinib failed to demonstrate an association with clinical benefit in patients with PBRM1 mutations in the atezolizumab monotherapy arm but instead favored benefit in the sunitinib arm [18]. A subsequent analysis from the Checkmate-025 study of patients with mRCC treated in the second-line or beyond and randomized to nivolumab or everolimus showed that there was enrichment of clinical benefit in the PBRM1 mutant group in nivolumab-treated patients, though this trial did not include a VEGF-targeted therapy. The effect of PBRM1 mutations on response and survival in this study was modest, with median PFS 5.6 vs. 2.9 months (HR, 0.67; 95% CI, 0.47–0.96; *p* = 0.03) and median OS 27.9 vs. 20.9 months (HR, 0.65; 95% CI, 0.44–0.96; *p* = 0.03) [34]. 

Finally, a large retrospective analysis (*n* = 2936) explored the interaction between PBRM1 mutations and immunotherapy across cancer types and failed to show a statistically significant association with OS (HR 0.9, *p* = 0.7). Interestingly, this trial included 189 patients with mRCC treated with ICB and this subgroup did demonstrate an association with OS (HR 1.24, *p* = 0.47). It was previously hypothesized in the initial discovery study by Miao et al. that PBRM1 mutations increased interferon-gamma (IFNγ) gene expression and thereby modulated the immune response. However, this analysis explored the impact of IFNγ signaling in both the cohort studied by McDermott et al. and a cohort from the previously mentioned COMPARZ trial and showed unchanged or decreased IFNγ signaling in PBRM1 mutants compared to the wild-type, which conflicted with the hypothesized mechanism of action [35]. Due to the conflicting nature of these results, doubt has been cast on the potential use of PBRM1 as a biomarker for ICB [36].

#### 3.2.2. TERT Promoter Mutations

Although much focus is in finding mutations associated with response, it is also useful to examine the opposite phenomenon and identify mutations that are associated with resistance to immunotherapy. This would help route patients to therapies more likely to be beneficial and avoid unnecessary toxicity. For example, in non-small cell lung cancer mutations in STK11 have been identified as predictors of poor responses to ICBs [37]. STK11 is not a useful biomarker for RCC since it is very rarely found in RCC on the order of 0.2% of patients based on data from cBioPortal [38]. A retrospective study of patients with mRCC (*n* = 75), the majority with clear cell histology (~80%), who received comprehensive genomic profiling (whole exome and RNA sequencing) as part of routine care, including both immunotherapy and targeted therapy, attempted to identify genomic and transcriptomic correlates of clinical benefit. The authors found that mutations in the TERT promoter were specifically associated with a lack of benefit from ICB. In this subgroup of TERT promoter mutated tumors the authors also found enrichment of transcription factor targets of MYC and KATA2, and kinase targets of CDK4, ATM, and MAPK14 [39].

#### 3.2.3. Multi-gene Expression Signatures

Similar to approaches in VEGF TKI treated patients, researchers have investigated potential tumor genomic signatures that might serve as predictive biomarkers for ICB. In an exploratory analysis of the IMmotion150 study, the authors used gene signatures previously defined and representing angiogenesis, immune response (T-effector/IFNγ), and myeloid inflammatory gene expression to perform a subgroup analysis and investigate associations with response. They found that tumors with high expression of a T-effector gene signature (T_eff_^High^) was positively associated with expression of PD-L1 and CD8 T-cell infiltration. They also showed that within this group there was increased expression of the myeloid inflammation genes. The T_eff_^High^ gene signature was also associated with improved ORR and PFS when compared to the T_eff_^Low^ group within the atezolizumab/bevacizumab arm. They also showed that T_eff_^High^ was associated with improved PFS when compared across groups to the sunitinib arm. High myeloid inflammation gene signature expression (Myeloid^High^), which had previously been shown to be associated with suppressed T-cell responses, was shown to be associated with worse PFS in both the atezolizumab monotherapy and atezolizumab/bevacizumab arms [18]. 

A separate group utilized machine learning techniques to build upon the prior IMmotion150 gene signatures to define a specific 66-gene signature created for mRCC using RNA sequencing data from The Cancer Genome Atlas (TCGA) dataset in cBioPortal. They identified that the genes in the IMmotion150 gene signature were selected by analysis of the literature and citations which defined the three biological axes explored in the study and not based on an empirical analysis of the data, which they considered to be a limitation of the previous approach. To develop their signature, they first leveraged the gene signatures defined by the IMmotion150 study to perform unsupervised clustering to categorize patients into three groups and confirmed they separated into the same three categories; angiogenesis, T-effector and myeloid inflammation. They then utilized a separate featured selection machine learning technique to analyze the global gene expression profile of the sub-classified patients and selected the top 500 ranked and subsequently refined them using several different techniques to investigate the underlying biology and came up with their 66-gene signature. Using training and validation cohorts, they were able to show that this signature performed better with regards to association with OS and DFS than the original IMmotion150 signature. However, interpretation of this signature thus far is limited since annotation of treatments record and outcome are not available in the TCGA data and survival data was calculated prior to the approval of ICBs. The signature does, however, hold promise to be tested in cohorts who did receive ICB to test what they hypothesize as an improvement in the clustering of patients into unique groups defined by tumor biology [40]. 

In an analysis of the results of KEYNOTE-427 (pembrolizumab monotherapy) 11 separate gene signatures were analyzed for associations with response. They identified one signature, a T-cell inflamed gene expression profile (GEP), which stood out demonstrating a strong association with ORR to pembrolizumab. The same T-cell inflamed GEP signature, however, was not associated with longer PFS or OS in the same study and thus remains hypothesis generating [41] (Table 2).

#### 3.2.4. DNA Damage Repair Mutations, Microsatellite Instability, and Tumor Mutational Burden

Although less common in some other tumor types, RCC can harbor alterations in DNA damage repair (DDR) pathways, including defects in DNA mismatch repair (dMMR). Loss of function of certain genes related to dMMR defects can lead to lead to high levels of microsatellite instability (MSI), which has been established as a biomarker for response to immunotherapy irrespective of tumor type [42]. MSI-Hi tumors are not a common finding in RCC and are estimated to be present in only 1–2% of cases [43]. As a result, MSI is not a practical biomarker in a broad sense for ICB in RCC since many non-MSI RCC tumors respond to immunotherapy. 

Looking more broadly, mutations in genes involved in the various DDR pathways, which do not necessarily result in MSI, are relatively prevalent in RCC. In one cohort published by Ged et al., about 19% of patients (43/229) with mRCC harbor DDR mutations, with CHEK2 and ATM being the most frequently mutated. In this cohort, they were able to demonstrate a correlation between DDR mutation status and superior OS (HR 0.41; 95% CI: 0.14–1.14; *p* = 0.09) in patients treated with ICB [44]. This finding was also reported in a smaller cohort (*n* = 34) by Labriola et al. who showed that patients with DDR mutant tumors had improved disease control (defined as CR, PR, or SD) with ICB [45]. 

Another measure of disruption of genomic integrity is tumor mutational burden (TMB). TMB is defined by the total number of non-synonymous alterations (single-nucleotide variants or insertions/deletions) and is typically calculated from next-generation sequencing (NGS) data of either the whole exome or large targeted panels. A high TMB is thought to be integral in promoting increases in the expression of tumor neoantigens which promote T-cell mediated immune responses against tumors [46,47]. TMB, similarly to MSI, has been investigated independent of tumor histology and has been shown to enrich response to ICB [48,49]. This also led to an FDA approval on 16 June 2020 of pembrolizumab for all TMB-high tumors (defined as >10 mutations per megabase) regardless of histology. 

However, this approval has been met with controversy because of concerns that cutoffs for TMB and its performance as a biomarker may differ between tumor types. This skepticism is supported in RCC based on some of the available data. For example, in the study discussed above by Labriola et al., which focused solely on RCC, there was no observed association between TMB and disease control in patients treated with ICB [45]. This was also seen in a separate and larger cohort of 592 patients treated with nivolumab (pooled analysis of checkmate 009, 010, and 025) showed no association with response to PD-1 blockade. Paradoxically, it also has been shown that high-TMB is actually associated with inhibition of immune cell infiltrates in RCC tumors, which supports and possibly explains these unexpected clinical observations on a cellular level [50].

Another interesting observation that may help explain why RCC is such an immunogenic tumor but has a characteristically low TMB is the distribution of mutations that comprise its TMB [51,52]. TMB high tumors traditionally have a predominance of many single nucleotide variants (SNVs) making up the majority of mutations, while RCC on the other hand has a uniquely high proportion of insertions and deletions (indels) relative to other tumors. This phenomenon was identified as part of an analysis of the Cancer Genome Atlas study of 5777 solid tumors which identified RCC tumors as having more than double the median proportion of indels to SNVs. The authors then hypothesized that indels are more efficient in the formation of immunogenic peptides serving as neoantigens and using in silico prediction models they were able to show an enrichment of high-affinity neoantigens from indels that was three times that of SNV [53]. This suggests that RCC may be a case of quality over quantity in regards to immunogenic mutations.

Another approach to improving performance of TMB as a biomarker is incorporating HLA correction. HLA correction is a computational method by which incorporation of loss of heterozygosity of HLA alleles is thought to improve upon TMB by predicting the proportion of functional neoantigens present. This has been studied in non-small cell lung cancer and shown to identify and reclassify tumors previously characterized as TMB-high and, in doing so, improve the association with the response to ICB, but has yet to be studied in RCC [54]. 

### 3.3. Analysis of Immune Cells

In the search for biomarkers predicting a response to ICB, the investigation has necessarily expanded beyond clinical- and tumor-dependent factors, such as performance status or genomics, and additionally focused on host-dependent components of the immune system. In order to study the cellular components of the immune system, including T-cells, neutrophils, NK cells, and antigen-presenting cells, a variety of techniques have been approached, ranging from simple analytes (like a complete blood count with differential) to more complex methods like flow cytometry and advanced staining techniques, like multiplex IHC.

#### 3.3.1. Neutrophil Lymphocyte Ratio

One of the first biomarkers developed to attempt to quantify the immunologic milieu in patients with cancer is the neutrophil lymphocyte ratio (NLR). The biologic rationale for this biomarker is that it is a representation of cancer-related inflammation indicating more aggressive disease. Calculated simply by dividing the absolute neutrophil count by the absolute lymphocyte count on a complete blood count, this biomarker is essentially free and readily available for all patients. The NLR has been studied specifically in mRCC and a high pre-treatment NLR has been shown to have both prognostic and predictive implications. However, data in the specific context of treatment with ICB is limited [55,56,57,58,59]. One retrospective study of NLR in 42 patients with mRCC treated with ICB demonstrated that a pre-therapy NLR < 3 was associated with longer PFS (HR, 2.937; 95% CI, 1.44 to 5.97; *p* = 0.003) and OS (pre-therapy: HR, 3.977; 95% CI, 1.23 to 12.89; *p* = 0.014) [60]. A subsequent and larger study of 142 patients with mRCC treated with ICB showed that lower baseline NLR was only associated with a trend toward lower ORR, shorter PFS, and shorter OS. In this study they also looked at NLR at six weeks and showed that it was a significantly stronger predictor of all three outcomes than baseline NLR. They also, interestingly, showed that a > 25% decrease in NLR from baseline to six weeks was associated with significantly improved outcomes in mRCC patients treated with ICB [61]. Both of these were relatively small studies and larger prospective trials are needed to validate these findings before they could be incorporated more commonly into clinical practice.

#### 3.3.2. Tumor Infiltrating Lymphocytes and Immune Microenvironment

Tumor infiltrating lymphocytes (TILs) and their role in the immune response against cancer has been investigated for decades and it is now understood that cytotoxic CD8+ T-cells, otherwise known as “T-killer cells,” are essential components of a robust anti-tumor immune response. TILs are activated when presented an antigen via the class I major histocompatibility complex and release cytotoxins to kill the targeted cells [62]. RCC has long been understood to be one of the most immune-infiltrated tumors, as suggested as long as 30 years ago in a study characterizing samples from 120 different tumor histologies [63]. However, mere infiltration of a tumor with TILs does not prove that these immune cells have been properly activated to mount an anti-tumor response. The presence of PD-1 positive immune cells has been associated with worse outcomes as these tumors with large populations of PD-1-positive immune cells have evolved to promote quiescence of the immune system, which allows tumors to avoid detection and explains their more aggressive prognosis [64]. It has also been shown that changes in PD-1-positive immune cells were observed in response to surgical resection in a study of peripheral blood mononuclear cells (PBMC) from 90 patients with RCC before and after nephrectomy. This study showed that increased PD-1 expression on CD14 bright myelomonocytic cells, effector T cells, and natural killer (NK) cells correlated to disease stage, and expression was significantly reduced on all cell types soon after surgical resection of the primary tumor. This further suggests the association between PD-1 positivity of immune cells and the pathophysiology of this disease [65]. 

This phenomenon was further investigated with the incorporation of various known immune cell surface markers to better characterize the specific phenotype of ineffective TILs and the associated immune microenvironment. Giraldo et al. classified tumors into three basic categories defined by the phenotypic characteristics of their immune cells: immune-regulated, immune-activated, and immune silent. They showed in their study of 40 patients with mRCC that the immune-regulated tumors displayed aggressive histologic features and a high risk of disease progression in the year following nephrectomy for localized disease. The immune-regulated phenotype in this study was defined by CD8+PD-1+TIM-3+Lag-3+ TILs and CD4+ICOS+ helper T-cells in the presence of CD25+CD127-Foxp3+/Helios+GITR+ Tregs [66].

Understanding the importance of the immune infiltrate and microenvironment, new research has focused on the relationship between immune cell surface markers and response to immunotherapy. In an analysis of Checkmate-025 by Braun et al., they confirmed that RCC tumors tend to be heavily infiltrated with CD8+ TILs, but did not show an association between highly infiltrated tumors and ICB response. Interestingly, they noted that highly infiltrated tumors were less likely to carry PBRM1 mutations, which may help explain the association between PBRM1 mutations and favorable prognosis in RCC [67]. In a subsequent analysis of Checkmate-025 by the same group, subsets of the immune infiltrate were classified with methods similar to those used by Giraldo et al. They were able to show that having high levels of CD8+PD1+TIM3-LAG3- TILs was associated with benefit from nivolumab. They also showed that there was a linear association with the increased density of these cells and improvement in ORR, PFS, and OS. Furthermore, these observations were not seen in everolimus-treated patients suggesting a specific relationship to ICB response [68]. This data suggests specifically that TIM3 and LAG3 are important additional checkpoints, since their presence on TILs is associated with reduced benefit from ICB targeting the PD-1 pathway only. There are several inhibitors in development for both LAG3 and TIM3, including a bispecific antibody which targets both PD-L1 and LAG-3 and may be able to improve responses in these patients who express resistant immune phenotypes [69,70].

Lastly, there has been new research exploring the role of cancer-associated fibroblasts (CAFs) and their role in modulating the microenvironment in several tumor types, including mRCC. In mRCC models, CAFs have been shown to recruit macrophages leading to remodeling of the tumor microenvironment and, via signaling through fibroblast activation protein-a (FAP), may promote more aggressive tumor behavior [71]. FAP expression has also been shown to be associated with sarcomatoid features in mRCC, which have been shown, in a subgroup analysis of IMmotion151, to benefit from ICB in the first-line setting with atezolizumab and bevacizumab as compared to the VEGF TKI, sunitinib [18,72]. Finally, in lung cancer models CAFs have been shown to induce PD-L1 expression which further implies a specific role in modulating the immune response [73]. While they are not implicated directly as potential biomarkers for immunotherapy, the importance of CAFs in the immune milieu suggests they may have importance in biomarker development or become a potential target for future immunotherapy strategies. 

## 4. Conclusions

Much progress has been made in the treatment of metastatic RCC since the early 2000s, first with the development of targeted therapies, and even more so with the addition of immunotherapy. Now, for the first time, clinicians are fortunate to have a dilemma of choice between equally efficacious first-line treatment for patients with this disease. Despite these advances, there is still ample room for improvement both in overcoming primary resistance and also in selecting the optimal treatment for each individual patient. Our review summarizes years of work and progress in both of these avenues but still few are validated for treatment selection with proven clinical utility. As a result, and in contrast to many other tumor types, there are still no biomarker-driven approvals for mRCC. However, we anticipate that as our understanding of the biology of mRCC and the molecular mechanisms that drive its evolution expands from studies like TRACERx Renal and large datasets, such as the TCGA, we will identify new biomarker-driven approaches to treatment [74]. Furthermore, as the treatment landscape of mRCC continues to evolve and more treatment options become available, the importance and need for clinically useful biomarkers will only increase. Given this growing need, we envision that a paradigm will be needed to guide clinicians to the best choice available to personalize treatment. 

## Figures and Tables

**Table 1:** Summary of biomarkers for angiogenesis inhibitors.

Biomarker	Key Findings as a Predictive or Prognostic Biomarker
von Hippel-Lindau (VHL) [7]	No correlation with patient outcome in general No correlation with ORR, PFS, or OS in patients treated with anti-VEGF therapy
Polybromo-1 (PBRM1) [11,12]	Associated with a longer duration of response to anti-VEGF therapy
SET domain containing 2, histone lysine methyltransferase (SETD2) [13]	No definite association with overall survival Does not predict response to sunitinib
BRCA1 Associated Protein 1 (BAP1) [19]	Associated with lower expression of angiogenesis-related genes Possibly blunts response to anti-VEGF therapy
Vascular Endothelial Growth Factor (VEGF) [14,15,16,21]	Intratumoral overexpression associated with worse OS but does not predict response to first-line sunitinib Lower ratio of soluble isoforms 121–165 (<1.25) may help to predict response to second-line sunitinib after progression on interferon-α Lower ratio of serum levels at the end of treatment to baseline level associated with longer PFS with first-line axitinib/pembrolizumab
Angiopoietins (Ang-1, Ang-2) [21]	Associated with longer PFS when treated with first-line axitinib/pembrolizumab when either of the following were observed: ○ Decrease in Ang-1 protein level at the end of treatment ○ Decrease in Ang-2 protein level mid-treatment
Angio gene signature (VEGFA, KDR, ESM1, PECAM1, ANGPTL4, and CD34) [18,19,20]	More often upregulated in VHL and PBRM1 mutants Increased expression correlated with higher ORR, PFS, and/or OS in patients treated with first-line sunitinib or pazopanib except when TP53 or BAP1 mutations were present Improved PFS with first-line avelumab/axitinib as compared to sunitinib monotherapy if the Angio expression level was low

**Table 2:** Summary of gene expression signatures.

Gene Signature	Dataset	Genes		Key Findings
**IMmotion150 Signature** [18]	**Sample size:** 263 patients **Study Type:** Randomized phase 2 study of atezolizumab alone or combined with bevacizumab (anti-VEGF) versus sunitinib	**Angiogenesis (Angio)**		T_eff_^High^ associated with PD-L1 expression and CD8 T-cell infiltration T_eff_^High^ vs. T_eff_^Low^ in atezolizumab + bevacizumab associated with improved ORR (49% vs. 16%) and improved PFS (HR 0.50; CI 0.30–0.86) T_eff_^High^ atezolizumab + bevacizumab vs. sunitinib improved PFS (HR 0.55; CI 0.32–0.95) Myeloid^High^ associated with worse PFS in immunotherapy arms Distinct population of Myeloid^High^ tumors within the T_eff_^High^ group T_eff_^High^Myeloid^High^ vs. T_eff_^High^Myeloid^low^ associated with worse activity of atezolizumab (HR 3.82; CI 1.70–8.60)
		VEGFA PECAM1 ANGPLT4 ESM1	FLT1 CD34 KDR	
		**Myeloid Inflammation**		
		CXCL1 CXCL2 CXCL3	CXCL8 IL6 PTGS2	
		**T-effector (T_eff_)**		
		CD8A CD27 IFNG GZMA GZMB PRF1 EOMES CXCL9 CXCL10 CXCL11	CD274 CTLA4 FOXP3 TIGIT IDO1 PSMB8 PSMB9 TAP1 TAP2	
**66 Gene Signature** [40]	**Sample Size:** *Training cohort* 469 patients *Validation cohort* 64 patients **Study Type:** Retrospective analysis of ccRCC patients from The Cancer Genome Atlas (TCGA)	**Angiogenesis**		T-effector genes clustered with Ca2+-flux Subclasified patients into 3 categories: Angio, T_eff_, and Mixed Mixed cohort expressed genes from all four pathways Angio cohort had improved survival compared to T_eff_ and Mixed (median OS 90.4 vs. 62.8 vs. 62.8 months) Angio cohort had better DFS as compared to T_eff_ (HR = 2.2091, *p* = 0.0201) and Mixed (HR = 1.7433, *p* = 0.0386) Not yet tested or validated in a cohort who was homogenously treated Developed on data prior to ICB
		VEGFA KDR EDNRB PECAM1 ANGPLT4 NOTCH1	EDN1 FLT1 CD34 STIM2 ESM1	
		**T-effector**		
		PSMB9 PSMB8 LTA SLA2 PYHIN1 PDCD1 EOMES	CTLA4 CD8A GZMB GZMA TIGIT PREF1	
		**Ca2+-flux**		
		CD2 CCL5 CCL4 GK2 LCK LAT	LCP1 CD38 LAX1 CD7 CD3E ITK	
		**Invasion**		
		XCL2 FOXP3 FERMT3 SLC9A3R2 FASLG NFATC1 CD72 WAS PTK2B CXCR3 CORO1A CCR5 PDE2A TBCA2R	FYB1 NES S1PR1 TCF4 HEY1 ETS1 PTPRB PPM1F MCF2L GJA1 VWF MYCT1 NOS3 IL16	
**T-cell Inflamed GEP** [41]	**Sample Size:** 78 patients **Study Type:** Open-label, single-arm phase 2 study of first-line pembrolizumab	**T-cell Inflamed**		T-cell-inflamed GEP associated with higher ORR No association with PFS or OS
		CXCR6 TIGIT CD27 LAG3 NKG7 STAT1 CD8A IDO1 CCL5	PSMB10 CMKLR1 CD274 (PD-L1) PDCD1LG2 (PD-L2) CXCL9 HLA.DQA1 CD276 HLA.DRB1 HLA.E	
